# The Cost-Effectiveness Analysis of an Integrated Mental Health Care Programme in Germany

**DOI:** 10.3390/ijerph19116814

**Published:** 2022-06-02

**Authors:** Annabel Sandra Mueller-Stierlin, Uemmueguelsuem Dinc, Katrin Herder, Julia Walendzik, Matthias Schuetzwohl, Thomas Becker, Reinhold Kilian

**Affiliations:** 1 Department of Psychiatry II, Ulm University, Bezirkskrankenhaus Günzburg, 89312 Günzburg, Germany; uemmueguelsuem.dinc@bkh-guenzburg.de (U.D.); katrin.herder@uni-ulm.de (K.H.); thomas.becker@medizin.uni-leipzig.de (T.B.); reinhold.kilian@uni-ulm.de (R.K.); 2 Institute of Epidemiology and Medical Biometry, Ulm University, 89075 Ulm, Germany; 3 Schön Klinik MVZ GmbH, 81541 München, Germany; jwalendzik@minddoc.de; 4 Department of Psychiatry and Psychotherapy, University Hospital Carl Gustav Carus Dresden, Technische Universität Dresden, 01307 Dresden, Germany; matthias.schuetzwohl@uniklinikum-dresden.de

**Keywords:** integrated care, assertive community treatment, mental illness, cost-effectiveness, health economics

## Abstract

The network for mental health (NWpG = Netzwerk psychische Gesundheit) is an umbrella association for non-medical community mental health care facilities across Germany which are enabled to provide multi-professional mental health care packages including medical and psychosocial services reimbursed by German statutory health insurances since 2009. The aim of this study is to analyse the cost-effectiveness of providing NWpG mental health care packages plus treatment as usual (NWpG) to treatment as usual alone (TAU) in Germany. In a prospective, multicenter, controlled trial over 18 months, a total of 511 patients (NWpG = 251; TAU = 260) were observed in five regions, four times at six-month intervals. The EQ-5D-3L and the Client Sociodemographic and Service Receipt Inventory (CSSRI) were used to estimate quality-adjusted life-years and total costs of illness. Propensity score-adjusted cost–utility analysis was applied using the net benefit approach. No significant differences in costs and QALYs between NWpG and TAU groups were identified. The probability of NWpG being cost-effective compared to TAU was estimated below 75% for maximum willingness to pay (MWTP) values between 0 and 125,000 EUR. The additional provision of the NWpG package is not cost-effective compared to TAU alone.

## 1. Introduction

Germany is one of the countries with the highest absolute and relative levels of expenditure on mental health care [1,2,3,4]. In contrast to international guidance, the German mental health care system is characterised by fragmented services provided by mental hospitals, general hospital-based psychiatric inpatient units, office-based psychiatrists or psychologists, and hospital-based outreach ambulatory services, and these are complemented by a range of non-medical vocational, residential and psychosocial services that are provided by vocational rehabilitation centres, community mental health care centres and residential facilities [5,6,7]. The threshold to access inpatient and other services is low in comparison with other countries. Evidence-based community-based integrated services such as Home Treatment and Crisis Resolution Teams, Assertive Community Treatment and Intensive Case Management Teams [8,9,10,11,12,13,14,15,16] are scarce. The historical development of the legal, financial, and structural framework of the German health care system is considered by experts to be the key reason for its inertia with respect to the implementation of innovative treatment concepts. The German federal social legislative code hampers the implementation of integrated services. While medical services are mainly reimbursed by statutory health insurance, social services are funded by local and regional social welfare agencies on the basis of taxation. A change of the social legislative code in 2009 allowed providers of community-based non-medical mental health care services to offer managed mental health care packages including medical and non-medical service components on the basis of capitated payment by statutory health insurances [17].

Meanwhile, about 80 community mental health care providers across Germany offer integrated mental health care programmes called “Netzwerk psychische Gesundheit” (NWpG), and about 10,000 patients per annum are enrolled in these programmes. The expectation was that integrated and team-based mental health care on a capitated payment basis would result in improved effectiveness and cost-effectiveness [18,19].

A prospective observational evaluation study indicated that NWpG programmes were not generally more effective than standard care with regard to clinical and non-clinical outcome indicators. Nevertheless, study participants enrolled in NWpG programmes experienced a higher level of shared decision making and were more satisfied with mental health care services than participants receiving standard care [20]. No cost-effectiveness data have yet been available for this programme. In this article, we present the health economic evaluation of the NWpG programme. Within this evaluation, the utilisation of health care services, the related health care costs from a societal perspective and the net monetary benefits using the change of quality-adjusted life-years were compared between patients enrolled in the NWpG programme and a control group of patients receiving standard care alone.

## 2. Materials and Methods

A prospective, multi-centre-controlled trial (trial registration DRKS00005111) was conducted, and outcomes of patients enrolled in the NWpG programme were compared with a control group of patients receiving standard care alone (treatment as usual, TAU). The NWpG programme was implemented in routine care prior to study onset with patients receiving treatment as usual (TAU) plus NWpG (NWpG). Therefore, random assignment of study participants to the treatment arm was not possible. A preference-based group allocation of 1:1 ratio was used and based on the type of care provision (NWpG vs. TAU) planned for the coming 18 months. Adults diagnosed with a mental illness (except for mental disorders due to physical conditions or psychoactive substance use) were eligible for inclusion in the trial. In detail, the following inclusion criteria were applied:

- Being between 18 and 80 years old;
- Being diagnosed with a mental illness of the ICD-10 categories F20–F69 and F91–F94 during the last 12 months;
- Having either had a psychiatric inpatient admission or an ambulatory prescription of antipsychotic, anxiolytic or antidepressant drugs during the last 12 months prior to enrolment;
- Not being eligible for receiving benefits from statutory long-term care insurance;
- Being a member of one of the NWpG-participating health insurances (only for the intervention group).

**Figure 1:**
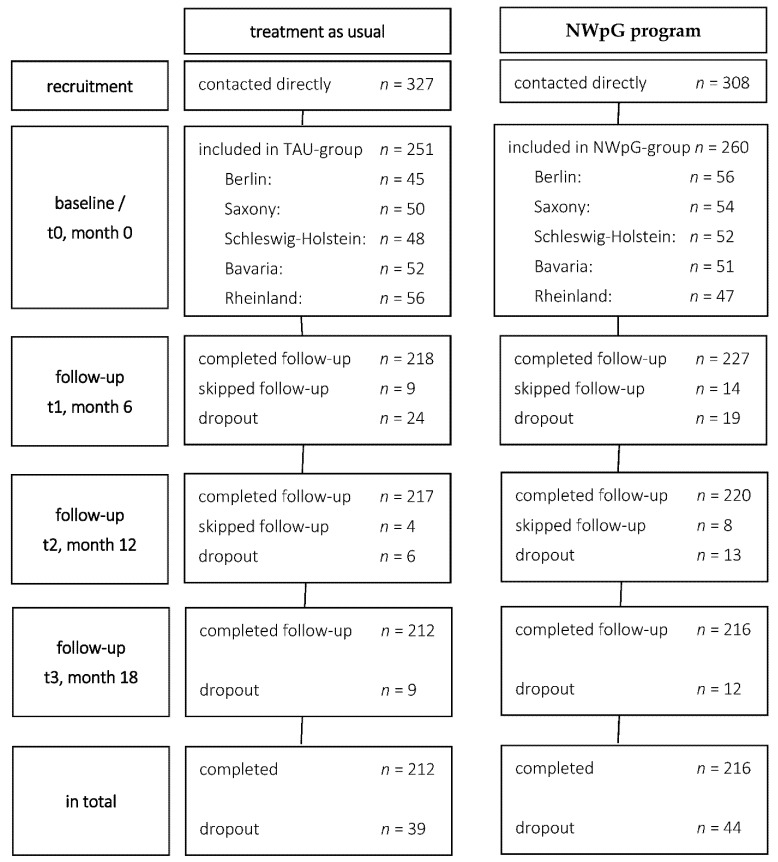
Flow of study participants: contacted directly—number of patients that were contacted and informed by study staff; completed follow-up—number of patients that completed follow-up (regardless of re-allocation); skipped follow-up—number of patients that skipped follow-up, but continued later on; dropout—number of patients who dropped out.

From August 2013 to January 2016, data were collected at baseline and at three follow-ups after 6, 12 and 18 months. Study sites were in five German federal states (Schleswig-Holstein, North Rhine-Westphalia, Berlin, Saxony, and Bavaria). An effect size of f = 0.2 was assumed to be clinically relevant for the within–between interaction of group time in repeated-measurements analysis of variance with two groups and four time-points of measurement. Based on this effect size, a power of 0.90, an alpha level of 0.05 and a drop-out rate of 30%, a sample size of >500 patients was the aim. A consecutive recruitment strategy was pursued. For the intervention group, patients enrolled in the NWpG programme during the last month were asked to participate. Using the same inclusion criteria as for NWpG enrolment, patients not willing to enrol in the NWpG programme or those who were not eligible for NWpG enrolment only due to their health insurance status were asked to participate in the control group (see Figure 1).

The study was conducted in compliance with the Declaration of Helsinki 2013. It was approved by the Ethics Committees of the University of Ulm (application number: 129/13) and of the TU Dresden (application number: EK 259072013). The protocol including details on design and sample size calculation was published in 2014 [21]. Details on the course of the IVPOWER study were reported in the main paper [20]. This manuscript follows the Consolidated Health Economic Evaluation Reporting Standards (CHEERS) [22].

### 2.1. Intervention

Participants in the intervention group received access to the integrated care services in the NWpG programme in addition to routine care. The NWpG programme comprises the coordination of community-based mental health care by mobile multi-professional teams. This includes case management, crisis intervention services and family-oriented psychoeducation. All relevant medical and social services are integrated and coordinated in close consultation with the service users and their families, or other persons to whom they feel close following an individually tailored mental health care plan [21].

Overall, no general superiority of NWpG over TAU was shown in the investigated primary and secondary outcomes (empowerment, clinical and psychosocial impairment, met service needs and quality of life). However, the study findings revealed a significantly greater improvement in terms of patients’ satisfaction with mental health care and their perception of treatment participation in the NWpG group compared to the control group [20].

### 2.2. Outcomes and Assessment

The health economic evaluation in the scope of the IVPOWER study focuses on the net benefit, using the change of quality-adjusted life-years and the change of service costs from a societal perspective.

The EQ-5D-3L was applied to assess generic health states on five dimensions (mobility, self-care, usual activities, pain/discomfort, and anxiety/depression), each with three levels of functioning (e.g., no problems, some problems, and extreme problems). Validity and responsiveness in assessing and valuing health status in patients living with mental illnesses were demonstrated by König et al. [23,24,25]. The German utility value set was used to generate quality-adjusted life-years (QALYs) [26,27].

The health economic analysis was carried out from a societal perspective. To this end, direct care costs and productivity losses resulting from sick leave times and times of disability pension were calculated. Since no central register of comprehensive costs of mental health care in Germany exists, the Client Sociodemographic and Services Receipt Inventory (CSSRI) was applied to collect individual information on medical and non-medical mental health services use on a self-report basis [28,29].

At all points of data collection (baseline, 6-, 12- and 18-month follow-up) service use, i.e., the means of frequency and duration of use were assessed (inpatient services received in the preceding six months, outpatient service use and non-medical service use during the preceding three months). Medication intake was quantified over the period of one month preceding data collection. In addition, data on housing situation, employment, social and insurance benefits (e.g., disability pension), were collected.

The CSSRI was adapted to be used in this study population in view of the mental health care settings at the participating study sites with special consideration given to the assessment of NWpG services. Frequency and duration of use of each type of service were extrapolated to a reference period of six months.

### 2.3. Cost Estimation

The half-yearly health care costs per trial participant (in Euros) were estimated based on the utilisation of health care services and respective unit costs. Given the short follow-up period of two years and the low-interest rates, the effect of discounting would be marginal and, therefore, no discount rates were applied. As there is no official list with unit cost data for services in Germany, data on current unit costs for the study period (2013 to 2016) were obtained from a variety of sources. Table S1 lists the key health services used by study participants and the corresponding unit costs. For some categories (e.g., inpatient and outpatient medical services), the unit costs are well-established [30]. For others (e.g., socio-psychiatric services, assisted living), unit costs were based on information by service providers and taken from previous trials [31]. The cost for outpatient pharmaceuticals was estimated on the basis of the number of days with drug intake during the past month and on the cost of defined daily doses of the respective drugs (DDD net cost, German Drug Prescription Report [32]). The NWpG programme was funded by an annual per-capita lump sum negotiated between the service providers and the health insurers. The respective lump sum is related to individual criteria for severity of illness and need for care. The service providers reported that the average lump sum, weighted for the number of participants at each study site, is 1485 EUR. The calculation was based on data of 5372 persons enrolled in NWpG between 2012 and 2015 who were insured by the health insurance provider (Techniker Krankenkasse) that initiated the NWpG programme.

Costs were adjusted for inflation from 2014 to 2021. The overall inflation rate for the time period was calculated at 10.3%.

### 2.4. Statistical Analysis

The analysis followed the intention-to-treat approach. Missing values due to loss to follow-up were imputed by the last observation carried forward (LOCF) method.

Propensity score (PS) adjustment was used to control for selection bias in the quasi-experimental setting [33]. PSs were estimated on the basis of a logistic regression model including baseline variables that showed limited balance between study groups (absolute standardised difference greater than 0.1) or that were associated (*p* < 0.10) with the primary outcome (change in empowerment score between baseline and third follow-up). Details of propensity score estimation including a complete variable list are published elsewhere [20].

Cost differences and 95% confidence intervals were estimated by means of linear regression models with robust standard errors computed by nonparametric bootstrapping with 1000 replications.

A seemingly unrelated regression (SURE) model [34] using feasible generalised least square estimation [35] was computed to estimate propensity score-adjusted cost and QALY differences and the correlation between both differences [36].

The incremental cost–utility ratio (ICUR) was estimated on the basis of PS adjusted cost and QALY differences [36,37]. Variance and confidence ellipse of the ICUR was estimated by means of Fieller’s theorem [36]. The probability of cost-effectiveness of NWpG (plus TAU) compared to TAU alone was estimated using the cost-effectiveness acceptability curve (CEAC) for a maximum willingness to pay (MWTP) range between 0 and 125,000 EUR. The net monetary benefit (NMB) of the NWpG intervention compared to TAU was estimated for an MWTP range between 0 and 125,000 EUR. All statistical analyses were conducted with STATA 17 [38]. ICUR variance, CEAC, and NMB were estimated using the STATA programme iprogs_0021.do provided by Glick [39].

## 3. Results

### 3.1. Study Population and Study Flow

In total, 511 patients (NWpG = 260, TAU = 251) were included and completed the data assessment at baseline, whereof 83 patients (16.2%) dropped out during 18 months of follow-up. Ten patients (4.0%) changed from the control group into the NWpG programme, and 22 patients (8.5%) in the NWpG group quit the programme after the baseline assessment (Figure 1).

The majority of study participants were female (353, 69.1%), and primarily diagnosed with depression (317, 62.0%). The average age was 46.5 years (*sd* 11.6). There are various indicators of a higher disease burden experienced over a longer time and associated with lower health and socio-economic status in the control group as compared with the NWpG group (Table 1).

### 3.2. Service Use

As indicated in Tables S2 and S3, slightly more than 10% of patients reported an inpatient stay in a psychiatric institution during the reference period, with that proportion being higher among TAU patients (13.5%) than in NWpG patients (10.0%). Approximately twice as many patients (21.9%) reported a somatic hospital stay, while half as many patients (5.3%) reported a psychiatric day hospital stay. Office-based specialists, psychiatric outpatient services or medical care centres were used by 87.9% of patients, with the frequency of use being about every other month. Three-quarters of patients (77.3%) had at least one contact with a general practitioner. The average frequency of contact with a general practitioner was two to three appointments in half a year. Other doctors were visited by more than half of the patients (58.5%), on average every second month. While almost two-thirds of NWpG patients received psychological treatment, that proportion was only 45% among TAU patients. One in seven patients underwent occupational therapy during the reference period.

Community psychiatric services such as day centres, contact and counselling centres and socio-psychiatric services were used by twice as many TAU patients as NWpG patients (TAU: 51.4%, NWpG: 23.5%). In addition, TAU patients attended these services more often (TAU: 3.2 days, NWpG: 1.1 days) and longer (TAU: 5.0 h, NWpG: 1.2 h) than NWpG patients.

Only seven TAU patients (2.8%) and one NWpG patient (0.4%) reported the use of crisis services or crisis apartments outside the NWpG programme. The utilisation rates for NWpG-specific crisis services were higher than for general crisis services (emergency telephone: 20.4%, crisis intervention team: 15.2%, crisis apartment: 4.8%, emergency appointment with psychiatrist 2.2%).

In general, a one-hour personal interview with NWpG staff took place every other month. Every eighth NWpG patient (12.3%) was visited at home by NWpG staff. In addition, two-thirds of all NWpG patients were in contact with NWpG staff every four months via telecommunications services (e-mail or telephone). Network meetings, i.e., meetings with the patients’ social and professional networks, were conducted with 35 patients (13.5%). NWpG group services were attended by one-tenth of the patients (10.0%).

The proportion of patients in assisted-living homes (TAU: 15.5%, NWpG: 4.2%) or in sheltered workplaces (TAU: 10.0%, NWpG: 5.8%) was higher in the TAU group than in the NWpG group.

Due to the higher prevalence of reduction in (up to the loss of) earning capacity in the TAU group (TAU: 37.5%, NWpG: 20.0%), the proportion of employed persons in this group was lower (TAU: 31.5%, NWpG: 60.0%). However, the monthly number of sick days among employed persons did not differ between the groups (TAU: 2.0, NWpG: 1.7).

### 3.3. Unadjusted Health Care Costs

Table 2 shows the unadjusted average annual health care costs with 95% confidence intervals for the 24-month study period. With the exception of somatic treatment costs, participants in the TAU group incurred significantly higher costs than those in the NWpG group. The overall cost difference amounts to −8096.92 EUR (95% CI (−11,735.75 EUR to −4458.10 EUR).

### 3.4. PS-Adjusted Health Care Costs, QALYs and Cost–Utility Ratios and Net Benefit

The results of the SURE model (Table S4) reveal that after adjusting for propensity scores, the annual cost difference reduces to −1183.04 EUR and is no longer significant (95% *CI* = −5270.27 to 2904.19). The adjusted QALY difference in the second part of the model is −0.0067, which is also not significant (95% *CI* = −0.047 to 0.033). A correlation of −0.24 between cost and QALY differences was estimated as the basis for the estimation of the ICUR confidence interval.

**Figure 2:**
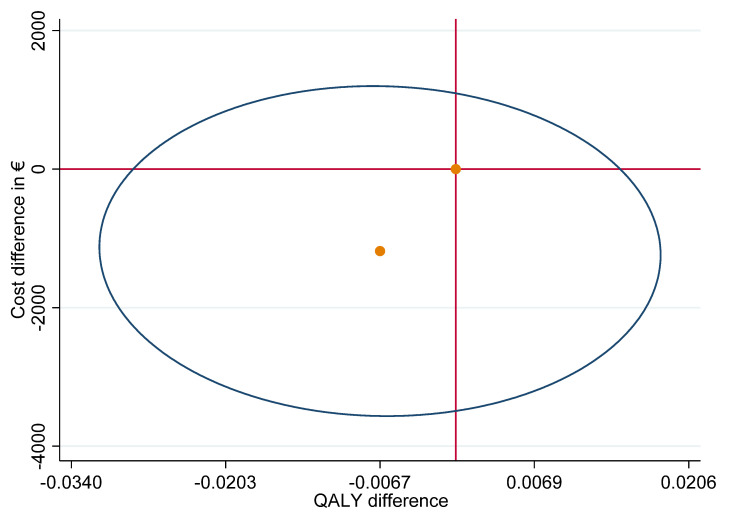
The location of the ICUR in the cost-effectiveness plane and the 95% variance ellipse (outer ellipse) of the ICUR variance.

The adjusted cost and QALY difference results in an ICUR of 176,573 EUR, which is located in the lower-left quadrant of the cost-effectiveness plane (CEP) (Figure 2) indicating that the NWpG intervention is less effective than TAU at lower costs. However, the estimation of the stochastic uncertainty revealed that the variance of the ICUR distributes over all quadrants of the CEP and that the estimation of a 95% confidence interval was not possible.

**Figure 3:**
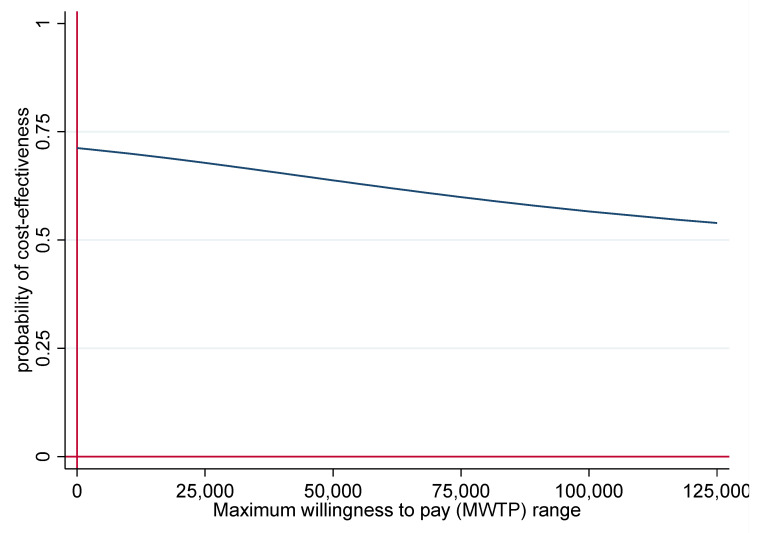
Cost-effectiveness acceptability curve indicating the probability of cost-effectiveness across the maximum willingness to pay range.

This result is supported by the cost-effectiveness acceptability curve (Figure 3), indicating that the probability of cost-effectiveness of the NWpG programme compared to TAU is below 75% for the MWTP range between 0 and 125,000 EUR. 

**Figure 4:**
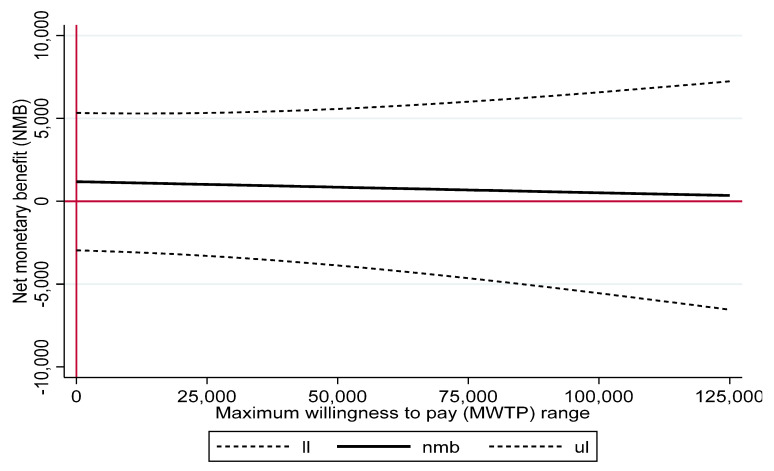
Net-monetary benefit regression curve. The solid black line indicates the net monetary benefit (nmb) in EUR which can be expected across the defined maximum willingness to pay range. The dotted lines indicate the upper and the lower limits of the 95% confidence interval of the nmb.

The inconclusiveness of the economic evaluation is also confirmed by the NMB regression curve (Figure 4) indicating that no significant net benefit can be expected in the defined MWTP range.

## 4. Discussion

The economic evaluation, after adjustment for sociodemographic and clinical differences between study groups, resulted in no significant difference in total costs or in cost-effectiveness between the NWpG integrated mental health care programme (plus TAU) and treatment as usual alone.

The current study was a pragmatic, non-blinded, multi-centre-controlled trial comparing outcomes among 260 persons living with mental illness enrolled in the NWpG programme with a control group of 251 patients who received standard care alone. Societal-perspective health care costs were estimated based on reported health service use in the previous months at baseline and at three follow-up assessments over a period of 18 months. We found that NWpG programmes were not generally more effective than standard care with regard to the primary outcome empowerment and other secondary outcomes. However, our study results suggested that the NWpG programme has the potential to increase treatment satisfaction and patients’ perceived treatment participation [20]. Second, we evaluated the use of common health services and of NWpG services, the associated health care costs, and the individual net benefit as part of the health economic evaluation.

As already discussed previously [20], the most important limitation of this study is the non-randomised assignment of study participants. Group differences were obvious, and it must be assumed that the propensity score adjustment may not have been able to remove the selection bias. Compared to patients in the control group, those in the NWpG group were less severely ill, their social status was higher and they were more socially connected [20]. These group differences are likely to be reflected in the lower utilisation of care services and lower health care costs in the NWpG group. Control group participants used more services with the exception of psychotherapy; the difference was particularly pronounced with regard to assisted living and social psychiatric care services. This leads to higher costs for participants in the control group, both in terms of direct costs, indirect costs, and total costs. Indirect costs account for two-thirds of the difference between the two study groups. However, the cost differences vanished after the PS adjustment.

Furthermore, the large variance in the duration of illness and the resulting heterogeneity of support needs could make it difficult to achieve a homogeneous treatment effect. In line with the effectiveness analysis for clinical and non-clinical outcomes [20], the programme was not shown to be effective in terms of EQ-5D-health states. As for the primary and secondary outcomes, our results fit with the findings of a systematic Cochrane review on intensive case management that revealed no evidence from randomised controlled trials for an overall improvement in mental health, social functioning or quality of life, but for treatment satisfaction [15].

In view of the lack of evidence of effectiveness in terms of both health outcomes and costs, the cost-effectiveness of NWpG was unlikely and this was verified by the incremental cost–utility analysis with cost and QALY differences adjusted for selection bias by means of propensity scores. Previous systematic reviews have reported inconsistent data on the utilisation of health services, associated costs and cost-effectiveness of similar interventions such as assertive community treatment [9] and intensive case management [15]. Cost-effectiveness was most likely to be observed among people with severe mental illness, e.g., schizophrenia-spectrum disorders [18,40,41], and particularly due to reduced hospital costs. A meta-regression showed that the higher the level of hospital utilisation at baseline, the greater the effect in reducing hospital use and inpatient cost [15]. Latimer concluded that hospital use prior to study inclusion should be about 50 days per year in order to break even with assertive community treatment [9]. That cut-off is substantially higher than the level of inpatient service use in the present study sample, where only 11.7% had been hospitalised during the preceding two-year period and the median duration of annual inpatient treatment was 23 days. This is probably also related to the fact that the severity of mental illness in the intervention group was moderate to low, with a small proportion of patients with a schizophrenia-spectrum disorder (9.2%).

As the level of NWpG service use was very low and a lump-sum case payment system was in place, the costs for individual appointments turned out to be very high. On average, there were only three regular contacts with NWpG staff in half a year, which would correspond to a daily rate of 250 EUR. However, it is important to keep in mind that service use data were collected from patient reports. This means that recall errors are possible and that the services provided in reserve, as well as unsuccessful attempts of contact, preparation and follow-up times, were not recorded. In addition, the cost estimate was based on a weighted rather than an individual lump sum, although in reality the lump sum is set according to the individual health state. Thus, no statement can be made about the appropriateness of the amount of the lump sum for NWpG. If a cost-neutral or cost-saving implementation of NWpG is aimed for, it would be necessary to compensate for the additional NWpG cost (amounting to the lump sum of 742.50 EUR in six months) by decreasing the duration of inpatient stays by slightly more than two days or by reducing the number of appointments in social psychiatric care institutions by 19 or more (in six months).

It cannot be ruled out that the study design chosen hampered the proof of cost-effectiveness. There were strong baseline differences between the NWpG and control group in this quasi-experimental trial, especially in terms of indirect costs due to work and disability and supported housing. The propensity score adjustment may not have been able to compensate for this selection bias. Moreover, it is challenging to achieve a reduction in indirect costs in only 18 months [42]. At the moment, the cost-effectiveness of integrated care approaches in Germany is being evaluated in two trials with target sample sizes of 1000 or 660 participants, respectively [43,44]. Given their study methodology, it may well be that the mentioned limitations were eliminated. These studies will expand our knowledge on to what extent integrated care approaches can be implemented cost-effectively in mental health services across the country.

## 5. Conclusions

To conclude, no health economic benefit of NWpG (plus TAU) compared to TAU alone was found. The IVPOWER study showed no evidence for effectiveness or cost-effectiveness, with the exception of secondary outcomes (satisfaction with and involvement in care). Further randomised trials would help. Adequate tailoring of integrated care interventions, a clear definition of target groups and robust strategies for implementing service innovation could be important in moving the field forward.

## Figures and Tables

**Table 1:** Sample characteristics at baseline.

	Total		TAU		NWpG		*p* ^a^
**Sociodemographic**							
Age in years; *m* (*sd*)	46.47	(11.61)	47.15	(11.01)	45.82	(12.14)	0.195
Female; *n* (%)	353	(69.1%)	167	(66.5%)	186	(71.5%)	0.221
Living alone; *n* (%)	235	(46.0%)	127	(50.6%)	108	(41.5%)	0.040
Employed; *n* (%)	175	(35.1%)	58	(23.5%)	117	(46.4%)	<0.001
Social welfare reception; *n* (%)	26	(5.1%)	20	(8.0%)	6	(2.3%)	0.004
Health insurance company affiliation, TK; *n* (%)	214	(41.9%)	32	(12.7%)	182	(70.0%)	<0.001
**Medical history and treatment**							
Duration of illness in years; *m* (*sd*)	12.47	(11.63)	14.24	(11.85)	10.77	(11.18)	0.001
Number of hospitalisations; *m* (*sd*)	2.89	(4.64)	4.06	(6.06)	1.77	(2.10)	<0.001
Diagnosis							0.017
F20–F29; *n* (%)	67	(13.1%)	43	(17.1%)	24	(9.2%)	
F30–F39; *n* (%)	317	(62.0%)	140	(55.8%)	177	(68.1%)	
F40–F48; *n* (%)	98	(19.2%)	53	(21.1%)	45	(17.3%)	
Multiple mental diagnoses; *n* (%)	248	(48.5%)	135	(53.8%)	113	(43.5%)	0.020
Prescription of pharmaceuticals; *n* (%)	379	(74.2%)	199	(79.3%)	180	(69.2%)	0.009
Assisted living; *n* (%)	36	(7.1%)	31	(12.4%)	5	(1.9%)	<0.001
Legal guardian; *n* (%)	31	(6.1%)	23	(9.2%)	8	(3.1%)	0.004
**Outcomes**							
Empowerment—EPAS total; *m* (*sd*)	3.42	(0.60)	3.42	(0.62)	3.42	(0.59)	0.959
Impairment—HONOS total; *m* (*sd*)	10.66	(5.30)	10.53	(5.12)	10.79	(5.48)	0.576
Number of needs; *m* (*sd*)	4.70	(2.63)	5.07	(2.70)	4.34	(2.51)	0.002
Proportion of met needs; *m* (*sd*)	59.5%	(31.8%)	62.9%	(30.4%)	56.2%	(32.8%)	0.017
Satisfaction score; *m* (*sd*)	24.22	(4.43)	24.51	(4.53)	23.94	(4.33)	0.154
WHOQOL-BREF; *m* (*sd*)	48.86	(22.04)	48.63	(22.64)	49.08	(21.49)	0.818
EQ-5D; *m* (*sd*)	0.77	(0.25)	0.74	(0.25)	0.79	(0.24)	0.028

^a^ Pearson Chi^2^ test for categorical and *t*-test for continuous variables.

**Table 2:** Average 12-month cost of illness over 24 months.

	Total	TAU	NWpG	Difference NWpG-TAU	*p*
	*M* (95% *CI* ^a^)	*M* (95% *CI* ^a^)	*M* (95% *CI* ^a^)	*M* (95% *CI* ^a^)	
Direct costs	9583.53	10,908.01	8288.25	−2619.76	0.002
	(8781.25 to 10,385.80)	(9488.82 to 12,327.19)	(7416.09 to 9160.42)	(−4276.02 to −963.49)	
Indirect costs	12,173.57	14,953.53	9448.72	−5500.42	<0.001
	(10,704.39 to 13,642.74)	(12,662.93 to 17,244.15)	(7526.98 to 11,370.43)	(−8350.15 to −2659.51)	
Total psychiatric costs (direct and indirect)	21,763.47	25,767.18	17,826,71	−7940.50	<0.001
	(19,940.70 to 23,586.24)	(11,455.41 to 14,311.79)	(15,607.98 to 20,045.44)	(−5762.91 to −2177.58)	
Somatic treatment	122.50	93.80	150.58	56.80	0.214
	(76.08 to 168.94)	(39.55 to 148.02)	(80.47 to 220.71)	(−32.83 to 146.41)	
Average 12-month total costs of illness over 24 months	21,997.82	26,111.33	18,014.39	−8096.92	<0.001
	(20,179.89 to 23,815.76)	(23,247.23 to 28,975.42)	(15,695.95 to 20,332.83)	(−11,735.75 to −4458.10)	

*M* = mean value of half-yearly health care costs in EUR, 95% *CI* = 95% confidence interval of half-yearly medical costs in EUR, *p* = significance measure for the cost difference NWpG minus TAU/significant group differences (*p* < 0.05) are printed in bold/^a^ non-parametric bootstrapping with 1000 replications.

## Data Availability

The datasets generated and/or analysed during the current study are not publicly available due to the used data protection declaration but are available from the corresponding author on reasonable request.

## Supplementary Materials

The following supporting information can be downloaded at: https://www.mdpi.com/article/10.3390/ijerph19116814/s1, Table S1: unit cost list; Table S2: description of utilisation of NWpG services; Table S3: description of utilisation of common health care services; Table S4: results of the seemingly unrelated regression (SURE) model for cost and QALY differences adjusted for propensity scores.
