# Supplementary material for: The Cost-Effectiveness Analysis of an Integrated Mental Health Care Programme in Germany
Source: Int J Environ Res Public Health. 2022 Jun 2;19(11):6814. doi: 10.3390/ijerph19116814 (PMC9180080; doi:10.3390/ijerph19116814)
Supplement: Supplementary file 1 [file ijerph-19-06814-s001.zip › ijerph-1684328-supplementary.pdf]

Table S1: unit cost list

Health care service	unit costs	
**Inpatient**		
Psychiatric hospital	339,71 €	per day
Somatic hospital	593,04 €	per day
Psychosomatic hospital	339,71 €	per day
Rehabilitation clinic	100,94 €	per day
**Day-care hospital**		
Psychiatric day-care hospital	220,81 €	per day
Somatic day-care hospital	385,48 €	per day
Psychosomatic day-care hospital	220,81 €	per day
**Outpatient**		
Medical care centre	44,72 €	per contact
Psychiatric outpatient clinic	70,00 €	for single contact
	270,00 €	for multiple contacts
**Practicioner**		
Psychiatrist	44,72 €	per contact
General practicioner	20,06 €	per contact
Other practicioner	38,63 €	per contact
Psychologist and Psychotherapist	90,00 €	per contact
Visiting nurse	29,92 €	per hour
**Complementary**		
Day care centre	55,00 €	per day
Socio-psychiatric services	39,00 €	per day
Crisis service	73,35 €	per hour
NWpG services	742,50 €	for six months
**Sonstiges**		
Occupational therapist	23,00 €	per hour
Nursing service	17,00 € + 4,76 €	per hour + travel costs
Housekeeping help	15,92 € + 4,76 €	per hour + travel costs
Physiotherapist	60,00 €	per hour
Caregiver	34,83 €	per hour
Voluntary caregiver	160,00 €	for six months
Assisted living	589,10 €	per month
Sheltered workplace	32,75 €	per day

Table S2: Description of utilization of NWpG services

	n	%	d	h
Regular contact (personal interview)	257	98.8%	0.44	0.50
Regular contact (by e-mail or telephone)	172	66.2%	0.25	-
Regular contact (at patient's home)	32	12.3%	0.10	0.13
Network conversation	35	13.5%	0.08	0.13
Group offers (including psychoeducation)	26	10.0%	0.58	0.84
Mediation of and accompanying to health care services	21	8.1%	0.08	0.08
Development of a crisis plan	4	1.5%	0.17	0.17
Emergency telephone	52	20.0%	0.22	0.06
Crisis intervention team	40	15.4%	0.16	0.17
Emergency appointment with psychiatrist	6	2.3%	0.15	0.05
Crisis apartment	12	4.6%	0.73	-

n = number of patients who used the care service in the reference period (six months before visit 1 to visit 4 (18 months after visit 1)), % = percentage of patients who used the care service in the reference period (six months before visit 1 to visit 4 (18 months after visit 1)), d = median of the monthly frequency of use in days (for users only), h = median of the frequency of use in hours in (for users only) / all patients who were enrolled in the NWpG program (including care group changers) were included in the analysis.

Table S3: Description of utilization of common health care services

	total				TAU				NWpG			
	n	**%**	**d**	**h**	***n***	**%**	**d**	**h**	***n***	**%**	**d**	**h**
Psychiatric hospital	60	11.7%	1.9	-	34	13.5%	1.9	-	26	10.0%	1.7	-
Psychosomatic hospital	18	3.5%	1.5	-	8	3.2%	1.2	-	10	3.8%	1.6	-
Rehabilitation clinic	29	5.7%	1.5	-	11	4.4%	1.5	-	18	6.9%	1.4	-
Psychiatric and psychosomatic day-care hospital	27	5.3%	2.7	-	13	5.2%	3.0	-	14	5.4%	2.6	-
Psychiatrists. psychiatric outpatient clinic. medical care centre	449	87.9%	0.6	0.2	236	94.0%	0.7	0.2	213	81.9%	0.5	0.2
General practicioner	395	77.3%	0.4	0.1	189	75.3%	0.4	0.1	206	79.2%	0.4	0.1
Other practicioner	299	58.5%	0.5	0.2	139	55.4%	0.5	0.2	160	61.5%	0.5	0.2
Psychologist and Psychotherapist	277	54.2%	1.4	1.2	113	45.0%	1.4	1.2	164	63.1%	1.4	1.2
Day care centre. socio-psychiatric services	190	37.2%	2.1	3.1	129	51.4%	3.2	5.0	61	23.5%	1.1	1.2
Occupational therapist	73	14.3%	1.4	1.7	41	16.3%	1.9	2.5	32	12.3%	1.2	1.4
Crisis service (including apartment)	8	1.6%	0.3	0.5	7	2.8%	0.3	0.8	1	0.4%	0.1	0.0
Assisted living	45	8.8%	4.0	5.0	36	14.3%	4.1	5.2	9	3.5%	2.2	2.7
Sheltered workplace	65	12.7%	1.6	2.6	40	15.9%	2.0	4.5	25	9.6%	1.1	2.0
Somatic hospital	112	21.9%	0.2	-	58	23.1%	0.2	-	54	20.8%	0.3	-

n = number of patients who used the care service in the reference period (six months before visit 1 to visit 4 (18 months after visit 1)), % = percentage of patients who used the care service in the reference period (six months before visit 1 to visit 4 (18 months after visit 1)), d = median of the monthly frequency of use in days (for users only), h = median of the frequency of use in hours in (for users only)

Table S4: Results of the seemingly unrelated regression (SURE) model for cost and QALY differences adjusted for propensity sores

Seemingly unrelated regression

Equation	Obs	Params	RMSE	"R-squared"	chi2	P>chi2
tcost12it~21	453	2	19644.85	0.1346	70.48	0.0000
qaly12itt	453	2	.1929575	0.0210	9.70	0.0078

	Observed coefficient	Bootstrap std. err.	z	P>|z|	Normal-based [95% conf.	interval]
tcost12ittinf21						
iv	-1183.037	2085.359	-0.57	0.571	-5270.266	2904.192
pst	-31369.13	4385.299	-7.15	0.000	-39964.16	-22774.1
_cons	38655.53	2181.284	17.72	0.000	34380.29	42930.77
qaly12itt						
iv	-.0066522	.020483	-0.32	0.745	-.0467982	.0334937
pst	.1246645	.0430737	2.89	0.004	.0402416	.2090874
_cons	.7338044	.0214252	34.25	0.000	.6918117	.775797

Correlation matrix of residuals:

	tcost12ittinf21	qaly12itt
tcost12ittinf21	1.0000	
qaly12itt	-0.2424	1.0000
